# Psychosocial burden and coping strategies among lymphoma patients: a nurse-led hematology care approach

**DOI:** 10.3389/fpsyg.2026.1783426

**Published:** 2026-05-04

**Authors:** Yan Wang, Tao Chen, Haolei Niu

**Affiliations:** Department of Hematology, The First Affiliated Hospital of Soochow University, Suzhou, Jiangsu, China

**Keywords:** coping strategies, HADS, lymphoma, nurse-led care, psychosocial distress, quality of life, supportive oncology

## Abstract

**Background:**

Patients with lymphoma frequently experience significant psychosocial burden, including anxiety, depression, and impaired quality of life, which may persist throughout the disease trajectory. Coping strategies and nurse-led care are recognized as important psychosocial determinants; however, their interrelationships remain insufficiently explored in lymphoma populations.

**Objective:**

This study sought to assess psychosocial distress, coping strategies, and quality of life in patients with lymphoma, while also investigating the influence of nurse-led care and the possible mediating effect of adaptive coping.

**Methods:**

A cross-sectional observational study was conducted among 200 patients with Hodgkin and non-Hodgkin lymphoma at a tertiary hospital in China. Psychological distress was assessed using the Hospital Anxiety and Depression Scale (HADS), quality of life using the EORTC QLQ-C30, and coping strategies using the Brief COPE Inventory. Nurse-led care was quantified using a composite nursing support score. Multivariable regression, mediation analysis, and receiver operating characteristic (ROC) analysis were performed.

**Results:**

Patients exhibited moderate levels of anxiety (9.6 ± 4.2) and depression (8.8 ± 3.9), with 45 and 37% of participants, respectively, scoring above the clinical cut-off (HADS ≥ 11). Global quality of life was moderately impaired (58.3 ± 16.5). Adaptive coping partially mediated the relationship between nursing support and quality of life. Higher nursing support scores were moderately and negatively correlated with both anxiety (*r* = −0.32, *p* < 0.001) and depression (*r* = −0.28, p < 0.001), and positively correlated with global quality of life (*r* = 0.27, *p* < 0.001). Nursing support demonstrated strong discriminative ability for clinically significant anxiety (AUC = 0.89).

**Conclusion:**

Lymphoma patients experience substantial psychosocial burden. Higher nursing support was associated with lower distress and better quality of life, partly through its correlation with adaptive coping strategies.

## 1 Introduction

Lymphoma represents a heterogeneous group of hematological malignancies arising from lymphoid tissues and accounts for approximately 4%–5% of all cancers worldwide. Recent global cancer statistics indicate that lymphoma remains a major contributor to cancer-related morbidity, with its prevalence steadily increasing due to improvements in early diagnosis, therapeutic efficacy, and long-term survival (Sung et al., 2021; Bray et al., 2012). Despite these advances, the growing population of lymphoma survivors has drawn increasing attention to non-malignant outcomes, particularly psychosocial sequelae that persist across the disease continuum (Hodgson et al., 2010).

Psychological distress, including anxiety and depressive symptoms, is highly prevalent among patients with lymphoma and has been consistently associated with impaired quality of life (QoL), reduced treatment adherence, and adverse clinical outcomes (Zabora et al., 2001; Linden and Vodermaier, 2012). Studies conducted in both newly diagnosed and long-term survivor populations have demonstrated that psychosocial distress may persist well beyond active treatment, reflecting ongoing disease-related uncertainty, fear of recurrence, and treatment-related toxicities (Lane et al., 2019; Emmanuel, 2023). Importantly, the magnitude of psychological burden appears to vary according to clinical characteristics such as disease stage, treatment modality, and comorbidity burden, underscoring the importance of individualized psychosocial assessment in hematology care (Valencia-Sanchez et al., 2021; Yamout et al., 2020).

Coping strategies constitute a central psychological mechanism through which individuals adapt to cancer-related stressors. Coping is broadly defined as the cognitive and behavioral efforts employed to manage internal and external demands perceived as exceeding available resources (Kavanagh, 2009). Within oncology populations, adaptive coping strategies such as acceptance, active coping, and problem-solving have been associated with improved psychological adjustment, greater resilience, and enhanced QoL (Stanton et al., 2000; Chan, n.d.). In contrast, maladaptive or avoidance-oriented coping strategies, including denial and behavioral disengagement, have been linked to heightened distress, poorer emotional functioning, and diminished well-being (Carver, 1997; Donovan-Kicken and Caughlin, 2011). Although coping has been extensively studied in solid tumor populations, empirical evidence specifically focusing on coping patterns among patients with lymphoma remains comparatively limited and fragmented.

Nurse-led care, in the context of this study, refers to a holistic, patient-centered model in which oncology nurses serve as the primary coordinators and deliverers of continuous psychosocial support, symptom management, patient education, and therapeutic communication throughout the cancer trajectory. This approach is grounded in an evidence-based, person-centred, and holistic framework in which nurses assume primary responsibility for care planning and delivery while collaborating with the multidisciplinary team. Nurses maintain the most frequent and sustained patient contact, enabling early identification of distress, facilitation of adaptive coping, and seamless integration of psychosocial support into routine hematology practice. Such nurse-led models have been shown to improve quality of life, reduce psychological distress, and enhance coping in cancer populations, including lymphoma and hematological malignancies (Furmenti et al., 2025; Young et al., 2020; Lam et al., 2020; Li et al., 2025; Zhang et al., 2025; Choi et al., 2025). This model emphasizes the unique position of nurses, who typically maintain the most frequent and sustained contact with patients, to identify early signs of distress, facilitate adaptive coping, and integrate psychosocial care into routine hematology practice (Lam et al., 2020; Oatley and Fry, 2020; Komatsu and Komatsu, 2023).

Unlike physician-centered or multidisciplinary team models, nurse-led care prioritizes nurse–patient relationship continuity, proactive psychosocial screening, and empowerment of patients through education and emotional support. This concept builds upon established frameworks such as the Oncology Nursing Society’s standards of care and patient-centered nursing models. In the present study, Nurse-led care was operationalized through a composite Nursing Support Score (detailed in the Methods section) to quantify its intensity and perceived quality.

Nurse-led care represents a cornerstone of comprehensive oncology is strongly associated with psychosocial outcomes in this population of patients with hematological malignancies (Lam et al., 2020; Oatley and Fry, 2020; Komatsu and Komatsu, 2023; Coulter and Oldham, 2016). Oncology nurses maintain continuous and close contact with patients throughout diagnosis, treatment, and follow-up, positioning them uniquely to identify psychological distress, provide emotional support, and facilitate adaptive coping responses (Thornton et al., 2020; Wender, 2020). Prior studies have demonstrated that nurse-led psychosocial interventions, structured patient education, and high-quality patient–nurse communication are associated with reduced psychological distress and improved QoL among cancer patients (Lam et al., 2020; Oatley and Fry, 2020; Thornton et al., 2020; Lancet Oncol., 2023). However, most existing studies have examined nursing interventions and psychosocial outcomes in isolation, without systematically evaluating the mechanisms through which nursing support may influence patient well-being.

Emerging conceptual frameworks suggest that coping strategies may serve as a key pathway linking supportive care interventions to psychosocial outcomes (Folkman and Moskowitz, 2000). Nevertheless, empirical investigations examining the mediating role of coping in the relationship between Nurse-led care and quality of life are scarce, particularly in lymphoma populations. Moreover, many prior studies lack an explicit nursing-focused analytical framework, limiting their translational relevance for clinical nursing practice in hematology settings.

Accordingly, the present study aimed to comprehensively evaluate psychosocial burden, coping strategies, and quality of life among patients with lymphoma using validated assessment instruments. Specifically, this study sought to (1) quantify levels of anxiety, depression, and quality of life; (2) examine associations between nurse-led care and psychosocial outcomes; and (3) explore the associations between nurse-led care, adaptive coping strategies, and quality of life. By adopting a nurse-led care perspective, this study aims to provide clinically relevant evidence to inform the development of targeted psychosocial nursing interventions and to optimize supportive care for patients with lymphoma.

## 2 Materials and methods

### 2.1 Study design and setting

**Figure 1:**
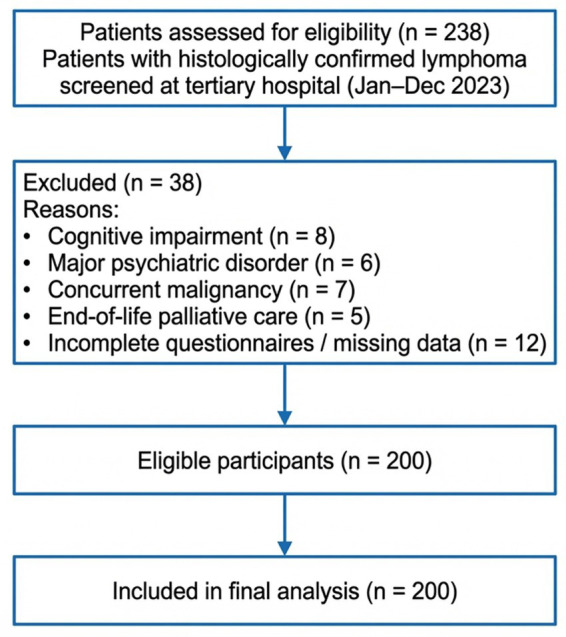
Participant recruitment and selection flow diagram. Flowchart illustrating the screening, eligibility assessment, exclusions, and final inclusion of patients with histologically confirmed lymphoma recruited from inpatient and outpatient hematology services between January and December 2023.

A cross-sectional observational study was conducted to evaluate psychosocial burden, coping strategies, and quality of life among patients with lymphoma, with particular emphasis on nurse-led hematology care. Participants were recruited from the hematology/oncology inpatient and outpatient services of a tertiary teaching hospital in Suzhou, Jiangsu Province, China, during the study period from January 2023 to December 2023 (Figure 1).

#### 2.1.1 Sample size determination

The sample size was determined based on established methodological recommendations for multivariable linear regression and receiver operating characteristic (ROC) analysis. For multiple linear regression models with continuous outcomes, a widely accepted rule of thumb recommends a minimum of 10–15 participants (observations) per predictor variable to promote model stability, minimize overfitting, and ensure reliable estimation of regression coefficients. More conservative guidelines, such as N > 50 + 8 m (where m is the number of predictors) for testing the overall model or N > 104 + m for individual predictors, have also been proposed. In the present study, the number of independent variables included in the fully adjusted regression models was deliberately limited (primarily disease stage, coping domain scores, nursing support score, and a small number of sociodemographic and clinical covariates), yielding a favorable participant-to-predictor ratio. With a final sample of 200 patients, this provided well above the recommended minimum, supporting stable estimation and reducing the risk of overfitting (Green, 1991).

For the exploratory ROC analysis, prior statistical guidance indicates that sample sizes of 150–200 participants are generally sufficient to estimate the area under the curve (AUC) with acceptable precision and confidence intervals. Due to the observational design and fixed one-year recruitment period, a formal *a priori* power analysis was not performed. Nevertheless, the achieved sample size of 200 provides sufficient statistical power to detect moderate effect sizes in the regression models and ensures reliable model stability, consistent with recommendations in psychosocial oncology and behavioral research (Jenkins and Quintana-Ascencio, 2020; Riley et al., 2019).

### 2.2 Participants and recruitment

Eligible participants were adults (≥18 years) with a histopathologically confirmed diagnosis of Hodgkin lymphoma or non-Hodgkin lymphoma. Patients were recruited using consecutive sampling at the point of routine clinical visit or hospitalization. All participants received standard-of-care hematologic treatment and routine nursing services within institutionally defined clinical pathways. These pathways provide a standardized evidence-based framework; however, care delivery including the frequency of nursing consultations and psychosocial interventions remains flexible and is tailored to individual patient needs, clinical status, and treatment phase.

#### 2.2.1 Inclusion and exclusion criteria

Patients were included if they (i) had confirmed lymphoma, (ii) were able to communicate effectively and complete questionnaires independently or with assistance from trained oncology nurses, and (iii) provided written informed consent. Patients were excluded if they had (i) severe cognitive impairment, (ii) a diagnosed major psychiatric disorder that could compromise the validity of self-reported outcomes, (iii) a concurrent active malignancy defined as any histologically confirmed second primary cancer (other than lymphoma) that was currently active, under active treatment, or not in complete remission at the time of enrollment, (iv) exclusive end-of-life palliative care status, or (v) substantial missing data in core psychosocial instruments precluding score computation.

### 2.3 Data collection procedures

Data were collected using standardized nursing assessment forms and validated self-report instruments. Trained oncology nurses introduced the study, obtained informed consent, and supervised questionnaire completion to ensure comprehension and minimize missing responses. Sociodemographic variables included age, sex, marital status, education level, and employment status. Clinical variables included lymphoma subtype, disease stage (I–II vs. III–IV), treatment modality (chemotherapy only; chemotherapy plus radiotherapy; immunochemotherapy), time since diagnosis, and comorbidity status. All nursing-related variables were extracted from standardized patient-level nursing records rather than individual nurse recall, ensuring accurate linkage of care encounters to each participant and minimizing recall bias.

## 3 Measures

### 3.1 Psychological distress (HADS)

Psychological distress was assessed using the Hospital Anxiety and Depression Scale (HADS), comprising two 7-item subscales for anxiety and depression (Zigmond and Snaith, 1983). Each item is scored on a 4-point scale (0–3), yielding subscale scores ranging from 0 to 21, with higher scores indicating greater symptom severity. In secondary analyses, clinically meaningful anxiety was operationalized as HADS-Anxiety ≥ 11, consistent with commonly used cut-offs in oncology populations.

### 3.2 Quality of life (EORTC QLQ-C30)

Quality of life was assessed using the European Organisation for Research and Treatment of Cancer Quality of Life Questionnaire Core 30 (EORTC QLQ-C30). Global health status/quality of life (Global QoL) scores were linearly transformed to a 0–100 scale following the EORTC scoring manual, with higher scores indicating better perceived global quality of life (Aaronson et al., 1993).

### 3.3 Coping strategies (brief COPE)

Coping strategies were assessed using the 28-item Brief COPE Inventory (Carver, 1997; Carver et al., 1989). Each subscale consists of two items rated on a 4-point Likert scale (1 = “I haven’t been doing this at all” to 4 = “I’ve been doing this a lot”), yielding subscale scores ranging from 2 to 8.

Following established theoretical frameworks and common practice in psycho-oncology research, coping strategies were grouped into three higher-order domains:

1. *Problem-focused (adaptive)*: Active coping, Planning, and Use of instrumental support. These strategies involve direct efforts to manage the stressor and are consistently associated with better psychological adjustment in cancer populations.
2. *Emotion-focused (generally adaptive)*: Emotional support, Positive reframing, Acceptance, Religion/spiritual coping, and Humor. These strategies aim to regulate emotional responses and are typically linked to improved quality of life when used in a flexible manner.
3. *Avoidance/maladaptive*: Denial, Behavioral disengagement, Substance use, Self-blame, Self-distraction, and Venting.

This classification is theory-driven rather than empirically derived from the current dataset (i.e., we did not use factor analysis on our sample to create domains). It is based on:

- Carver’s original conceptual model and subsequent validations of the Brief COPE;
- Meta-analyses and oncology-specific studies showing that avoidance-oriented strategies (denial, disengagement, self-blame, substance use) are reliably associated with higher distress and poorer adjustment;
- Consistent findings that self-distraction and venting, while not always overtly harmful, frequently correlate with increased emotional distress in cancer patients because they may impede active processing of the illness experience or prolong rumination.

Self-distraction and venting were classified within the avoidance/maladaptive domain because, in the context of a life-threatening illness such as lymphoma, these strategies have been shown in multiple studies to be associated with higher anxiety, depression, and lower quality of life. However, we acknowledge that both strategies can be adaptive in certain acute situations or cultural contexts. Therefore, we also conducted sensitivity analyses treating self-distraction and venting as separate “mixed” strategies; the overall pattern of results remained unchanged. Domain scores were calculated by averaging the standardized subscale scores within each category. Higher domain scores indicate greater reliance on the respective coping approach (Folkman and Moskowitz, 2000).

### 3.4 Nurse-led care/nursing support

Nurse-led care was operationalized through a self-designed composite Nursing Support Score (NSS) developed specifically for this study. The NSS quantitatively captures the intensity and perceived quality of nursing engagement within a patient-centered framework, consistent with established nurse-led supportive care models in oncology (Li et al., 2025).

#### 3.4.1 Construction method

The NSS was developed through a three-step process:

1. Literature review of oncology nursing standards and existing supportive care indices.
2. Consultation with experienced oncology nurses (*n* = 8) and psycho-oncologists (*n* = 3) to identify the most relevant and feasible indicators of nursing support in the Chinese hematology context.
3. A small pilot study (*n* = 25 lymphoma patients, conducted November–December 2022, not included in the main analysis) to test feasibility, clarity, and preliminary psychometric properties. Minor wording adjustments were made based on pilot feedback.

Internal consistency of the final Nursing Support Score was good (Cronbach’s *α* = 0.82). Content validity was established through expert panel review. Eleven experts (8 senior oncology nurses and 3 psycho-oncologists) rated the relevance of each NSS component and item to the construct of nurse-led supportive care in hematology settings on a 4-point ordinal scale (1 = not relevant to 4 = highly relevant). The Item-level Content Validity Index (I-CVI) was computed as the proportion of experts giving a rating of 3 or 4 for each item. The Scale-level Content Validity Index using the averaging method (S-CVI/Ave) was calculated as the mean of all I-CVIs. All I-CVIs were ≥ 0.82, and the S-CVI/Ave reached 0.91, indicating excellent content validity. Face and content validity were further supported by the expert panel’s qualitative feedback and minor wording adjustments made following the pilot study (*n* = 25). Construct validity was supported by significant correlations in the expected directions with psychological distress (negative) and quality of life (positive) in the current sample. As this is a newly developed pragmatic index, further external validation in independent and multi-center cohorts is warranted (Polit and Beck, 2006; Polit et al., 2007).

Although all patients were managed within standardized clinical pathways, these pathways are inherently adaptive and allow for individualized nursing care. Consequently, variability in the frequency of nursing encounters and the range of psychosocial interventions reflects patient-specific needs and clinical complexity rather than inconsistency in care delivery. The components of the NSS were designed to capture distinct but complementary dimensions of nurse-led care. Specifically, the frequency of nursing consultations reflects the intensity of patient–nurse contact (quantitative exposure), whereas the psychosocial intervention component reflects the content and breadth of therapeutic supportive care delivered (qualitative input). These dimensions are conceptually independent; a higher frequency of encounters does not necessarily imply delivery of structured psychosocial interventions, and vice versa. Including both components allows a more comprehensive assessment of nurse-led care within a patient-centered framework.

#### 3.4.2 Main components and items

The NSS comprises three complementary components:

##### 3.4.2.1 Frequency of nursing consultations (nurse-documented, objective)

Number of documented face-to-face or telephone nursing encounters per patient per month, derived from standardized nursing records. In routine clinical practice, patients are often cared for by multiple nurses. To ensure accurate attribution, encounter data were extracted at the patient level from nursing documentation systems using each patient’s unique medical record number. Thus, the measure reflects the total number of nursing encounters received by each patient, irrespective of the individual nurse providing care.

Given the cross-sectional design, this variable was calculated retrospectively:

- For hospitalized patients, encounters were counted during the current hospitalization period and standardized to a monthly rate.
- For outpatients or patients with longer disease duration, encounters since diagnosis were aggregated and converted into an average number of encounters per month.

This approach avoids reliance on recall and ensures consistency with cross-sectional observational methodology. To reduce the influence of extreme values and variability in care intensity, the monthly encounter count was capped at 12 encounters.

This cap was introduced as a statistical normalization strategy rather than a clinical constraint. Its purpose was to reduce the influence of extreme values (e.g., patients with prolonged hospitalization or unusually intensive monitoring) that could disproportionately affect composite score distribution and regression estimates. The cap reflects a level at which nursing contact frequency reaches a practical saturation point in routine hematology care, as informed by pilot data and clinical consultation. All raw encounter data were collected without restriction, and the cap was applied only during score standardization. Sensitivity analyses using uncapped values yielded consistent results, supporting the robustness of the findings.

The raw monthly encounter value was transformed to a 0–10 scale using the formula:

Item 1 score = [min(raw value, 12)/12] × 10.

where 12 represents the predefined upper cap applied for statistical normalization.

##### 3.4.2.2 Receipt of psychosocial nursing interventions (nurse-documented, objective)

Number of distinct psychosocial and supportive care interventions delivered by oncology nurses during the treatment episode, derived from standardized nursing records. Interventions were categorized into predefined domains (e.g., emotional support, symptom management education, stress-reduction techniques, family communication facilitation), with a total possible range of 0–8 categories. This approach captures the breadth and diversity of nursing interventions, rather than the frequency of repeated delivery of the same intervention. Repeated instances of the same intervention type were counted once to avoid overrepresentation. The bounded range reflects the predefined taxonomy of intervention categories and should not be interpreted as a restriction on actual clinical practice.

The raw score for Item 2 was defined as the number of distinct types of psychosocial nursing interventions received by each patient, based on predefined categories documented in nursing records.

The intervention categories included:

- Emotional support/counseling
- Symptom management education
- Stress-reduction techniques (e.g., relaxation, mindfulness)
- Facilitation of family communication
- Referral to psycho-oncology services
- Treatment-related education
- Coping skills guidance
- Other structured supportive interventions

Each category was counted once per patient, regardless of how many times it was delivered, to reflect the breadth of interventions rather than repetition frequency. The raw score therefore ranged from 0 to 8 categories.

The raw intervention count was transformed to a 0–10 scale using the formula:

Item 2 score = (raw value/8) × 10

##### 3.4.2.3 Perceived patient–nurse communication quality (patient-reported, subjective)

- Three items adapted from validated communication tools (EORTC QLQ-INFO25 and Picker Patient Experience Questionnaire):
- “How clearly did the nurses explain things in a way you could understand?”
- “How often did nurses show respect and listen carefully to your concerns?”
- “How satisfied were you with the emotional support provided by the nursing team?”
- Each item rated on a 5-point Likert scale (1 = poor to 5 = excellent), summed (range 3–15).

The communication quality component (3 items, Likert scale 1–5 each) was summed (range 3–15) and standardized as:

Item 3 score = [(raw sum—3)/12] × 10.

#### 3.4.3 Final scoring method

The three standardized component scores were averaged with equal weighting:

NSS = (Item 1 score + Item 2 score + Item 3 score)/3.

Yielding a final score ranging from 0 to 10, with higher scores indicating greater overall nursing support.

#### 3.4.4 Reliability and validity

The psychometric properties of the Nursing Support Score (NSS) were evaluated in terms of internal consistency, content validity, and construct validity.

*Internal consistency*: The NSS demonstrated good reliability, with a Cronbach’s *α* of 0.82, indicating satisfactory coherence among its components.

*Content validity*: Content validity was established through expert panel evaluation. Eleven experts (8 senior oncology nurses and 3 psycho-oncologists) independently rated the relevance of each NSS component and item using a 4-point ordinal scale (1 = not relevant to 4 = highly relevant). The Item-level Content Validity Index (I-CVI) for all items was ≥ 0.82, and the Scale-level Content Validity Index (S-CVI/Ave) was 0.91, indicating excellent content validity. Minor wording refinements were made based on expert feedback and pilot testing (*n* = 25) to enhance clarity and applicability.

*Construct validity*: Construct validity was supported by statistically significant associations in the expected directions between the NSS and key psychosocial outcomes. Higher NSS scores were associated with lower levels of anxiety and depression and higher quality of life, consistent with theoretical expectations of nurse-led supportive care.

As the NSS is a newly developed pragmatic composite index, further external validation in independent and multi-center cohorts is warranted.

### 3.5 Study outcomes

The primary outcomes were levels of psychosocial burden (HADS-Anxiety, HADS-Depression) and global quality of life (EORTC QLQ-C30). Secondary outcomes included coping strategy patterns and their associations with nursing support. An exploratory objective was to evaluate the discriminative ability of the Nursing Support Score for identifying patients with clinically significant anxiety (HADS-Anxiety ≥ 11). This analysis was conducted because oncology nurses have frequent patient contact and routinely document care intensity; therefore, we explored whether a composite nursing support metric could serve as a pragmatic risk-stratification indicator to help prioritize patients who may benefit from more formal psychological assessment. The NSS was not developed or evaluated as a standalone diagnostic or screening tool.

### 3.6 Statistical analysis

Analyses were performed using IBM SPSS Statistics (version 28.0; IBM Corp., Armonk, NY, United States) and R (version 4.3.1; R Foundation for Statistical Computing, Vienna, Austria). Continuous variables were summarized as mean ± standard deviation or median (interquartile range), as appropriate; categorical variables were summarized as frequency (percentage). Distributional assumptions were evaluated using visual inspection and normality testing. Between-group comparisons (e.g., early vs. advanced stage) used independent-samples t-tests or Mann–Whitney U tests for continuous variables and chi-square tests for categorical variables; multi-group comparisons used ANOVA or Kruskal–Wallis tests with suitable *post hoc* procedures.

Bivariate associations between nursing support, psychosocial outcomes, and coping variables were assessed using Pearson or Spearman correlation coefficients depending on distributional properties. Multivariable regression models were used to identify independent predictors of psychosocial distress and quality of life. Hierarchical (blockwise) modeling was employed to quantify incremental explanatory power across sequential models: Model 1 (unadjusted), Model 2 (adjusted for sociodemographic variables), and Model 3 (fully adjusted for clinical characteristics and nurse-led care variables). Multicollinearity was assessed using variance inflation factors.

A mediation analysis was conducted to evaluate whether adaptive coping mediated the association between nursing support and quality of life. Indirect effects were tested using nonparametric bootstrapping (5,000 resamples) with bias-corrected confidence intervals; mediation was considered present if the bootstrap confidence interval for the indirect effect excluded zero. Receiver operating characteristic (ROC) analysis was performed as an exploratory analysis to assess the performance of the Nursing Support Score in discriminating clinically significant anxiety. The area under the curve (AUC) and 95% confidence intervals were calculated, and the optimal cut-off was determined using the Youden index. We emphasize that this analysis was intended to explore the potential utility of nursing-documented information for risk stratification only, and that the NSS should not replace validated psychological screening instruments such as the HADS. All tests were two-sided, and statistical significance was set at *p* < 0.05.

### 3.7 Ethical considerations

The study protocol was reviewed and approved by the Institutional Review Board of the participating hospital (Department of Hematology, The First Affiliated Hospital of Soochow University). The study was conducted in accordance with the Declaration of Helsinki. All participants provided written informed consent prior to participation. Data were anonymized prior to analysis, and confidentiality was maintained throughout the study.

## 4 Results

### 4.1 Participant characteristics

A total of 200 patients with histologically confirmed lymphoma were included in the final analysis. The mean age of participants was 52.4 ± 13.6 years, with a predominance of male patients (56.0%). Most participants were married (73.0%), and approximately one quarter had attained higher education. Regarding clinical characteristics, 69.0% of patients were diagnosed with non-Hodgkin lymphoma, and 61.0% presented with advanced-stage disease (stage III–IV). Chemotherapy alone was the most common treatment modality (52.0%), followed by combined chemotherapy and radiotherapy (34.0%). Detailed sociodemographic and clinical characteristics are summarized in Tables 1, 2.

**Table 1:** Sociodemographic characteristics of patients with lymphoma (*n* = 200).

Variable	Category	*n* (%)
Age (years)	Mean ± SD	52.4 ± 13.6
	< 40	48 (24.0)
	40–59	82 (41.0)
	≥ 60	70 (35.0)
Sex	Male	112 (56.0)
	Female	88 (44.0)
Marital status	Married	146 (73.0)
	Single/Widowed/Divorced	54 (27.0)
Education level	Primary or below	64 (32.0)
	Secondary	86 (43.0)
	Higher education	50 (25.0)
Employment status	Employed	94 (47.0)
	Unemployed/Retired	106 (53.0)

This table summarizes the baseline sociodemographic characteristics of the study population, including age, sex, marital status, education level, and employment status. Continuous variables are presented as mean ± standard deviation, and categorical variables are presented as number (percentage).

**Table 2:** Clinical characteristics of patients with lymphoma (*n* = 200).

Variable	Category	*n* (%)
Lymphoma subtype	Hodgkin lymphoma	62 (31.0)
	Non-Hodgkin lymphoma	138 (69.0)
Disease stage	Stage I–II	78 (39.0)
	Stage III–IV	122 (61.0)
Treatment modality	Chemotherapy only	104 (52.0)
	Chemotherapy + radiotherapy	68 (34.0)
	Immunochemotherapy	28 (14.0)
Time since diagnosis	< 6 months	72 (36.0)
	≥ 6 months	128 (64.0)
Comorbidities	None	88 (44.0)
	≥ 1 comorbidity	112 (56.0)

Clinical characteristics of the study participants, including lymphoma subtype, disease stage, treatment modality, time since diagnosis, and comorbidity status. Values are presented as number (percentage).

### 4.2 Psychosocial burden and quality of life

Overall, patients demonstrated moderate levels of psychological distress. The mean HADS-Anxiety score was 9.6 ± 4.2, and the mean HADS-Depression score was 8.8 ± 3.9. Using the established clinical cut-off of HADS subscale score ≥ 11, 45% (*n* = 90) of patients had clinically significant anxiety and 37% (n = 74) had clinically significant depression. The mean Global Quality of Life score assessed by the EORTC QLQ-C30 was 58.3 ± 16.5, indicating moderate impairment in perceived well-being **(**Table 3**)**. Based on established cut-off criteria, a substantial proportion of patients met thresholds indicative of clinically relevant anxiety and depressive symptoms. These prevalence rates underscore the substantial psychosocial burden in this lymphoma cohort and highlight the need for routine screening.

**Table 3:** Psychological distress and quality of life scores among patients with lymphoma.

Measure	Mean ± SD	Range	Clinically significant* *n* (%)
HADS–anxiety score	9.6 ± 4.2	0–21	90 (45.0%)
HADS–depression score	8.8 ± 3.9	0–21	74 (37.0%)
EORTC QLQ-C30 global QoL	58.3 ± 16.5	0–100	—

*HADS subscale score ≥ 11 was used to define clinically significant anxiety and depression, consistent with recommended cut-offs in oncology populations. Mean scores and ranges for anxiety and depression assessed by the HADS and global quality of life assessed by the EORTC QLQ-C30 questionnaire. Higher HADS scores indicate greater psychological distress, whereas higher EORTC QLQ-C30 scores indicate better quality of life.

### 4.3 Coping strategy profiles

Analysis of coping strategies revealed that adaptive coping mechanisms were more frequently utilized than maladaptive strategies. Among problem-focused approaches, active coping (6.1 ± 1.4) and planning (5.8 ± 1.6) were commonly reported. Within emotion-focused coping, acceptance emerged as the most highly utilized strategy (6.3 ± 1.3), followed by emotional support (5.9 ± 1.5). In contrast, avoidance-oriented coping strategies such as denial (3.4 ± 1.5), behavioral disengagement (3.1 ± 1.4), and substance use (2.6 ± 1.2) were infrequently reported **(**Table 4**)**.

**Table 4:** Distribution of coping strategies assessed by the brief COPE inventory.

Coping domain	Coping subscale	Mean ± SD	Interpretation
Problem-focused coping	Active coping	6.1 ± 1.4	Frequently used adaptive strategy
	Planning	5.8 ± 1.6	Moderate–high engagement
	Use of instrumental support	5.4 ± 1.7	Regular reliance on practical assistance
Emotion-focused coping	Emotional support	5.9 ± 1.5	Commonly used
	Positive reframing	5.2 ± 1.6	Moderate adaptive coping
	Acceptance	6.3 ± 1.3	Highly utilized
	Religion/spiritual coping	4.8 ± 1.9	Variable across patients
	Humor	3.9 ± 1.8	Less frequently used
Avoidance/maladaptive coping	Self-distraction	5.1 ± 1.6	Moderately present
	Denial	3.4 ± 1.5	Low utilization
	Behavioral disengagement	3.1 ± 1.4	Low utilization
	Substance use	2.6 ± 1.2	Rarely reported
	Self-blame	4.2 ± 1.7	Moderate maladaptive tendency
	Venting	4.6 ± 1.8	Moderate emotional release

Mean scores for problem-focused, emotion-focused, and avoidance-oriented coping strategies as measured by the Brief COPE Inventory. Higher scores reflect more frequent use of the corresponding coping strategy.

### 4.4 Associations between nursing support and psychosocial outcomes

**Figure 2:**
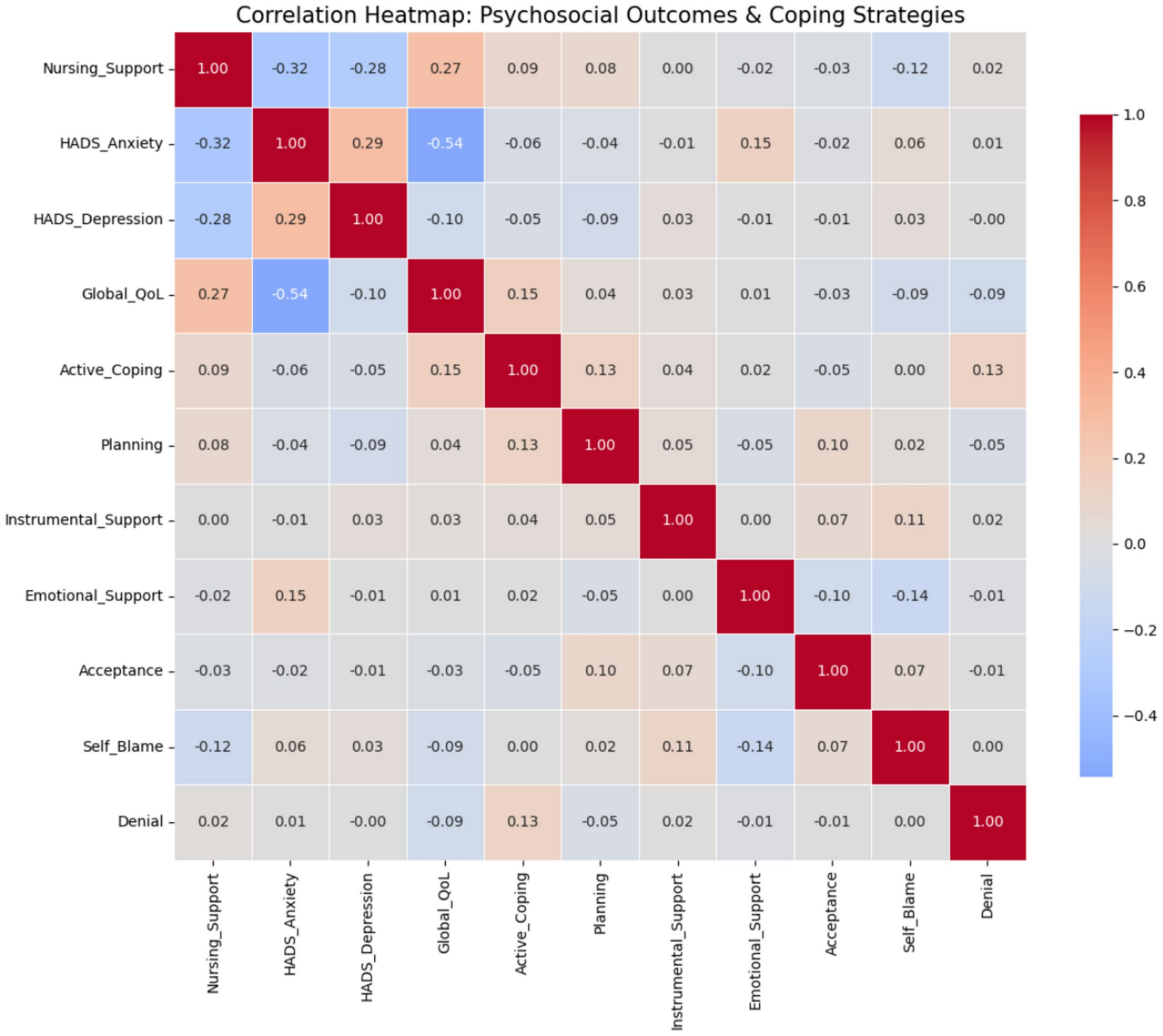
Correlation heatmap of nursing support, psychosocial outcomes, and coping strategies. Heatmap illustrating pairwise correlations among nurse-led care, psychological distress (HADS anxiety and depression), quality of life, and coping strategy domains. Color intensity reflects the strength and direction of correlations.

**Figure 3:**
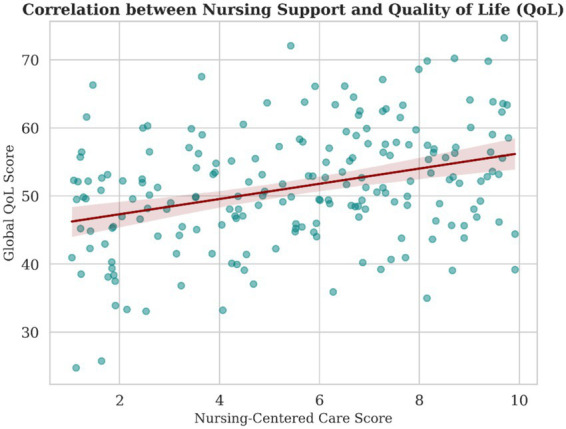
Association between nurse-led care and quality of life. Scatter plot depicting the relationship between nursing support score and global quality of life (EORTC QLQ-C30). The solid line represents the fitted linear regression line, with shaded areas indicating 95% confidence intervals.

Bivariate correlation analysis demonstrated significant associations between nurse-led care and key psychosocial outcomes **(**Table 5**)**. Higher nursing support scores were moderately and negatively correlated with HADS-Anxiety (*r* = −0.32, *p* < 0.001) and HADS-Depression (*r* = −0.28, *p* < 0.001), and positively correlated with global quality of life (*r* = 0.27, *p* < 0.001), as illustrated in Figures 2, 3. The positive linear association between nursing support and global quality of life is illustrated in Figure 3. In addition, nursing support showed weak but positive correlations with adaptive coping strategies, supporting further multivariable and mediation analyses.

**Table 5:** Associations between nurse-led care and psychosocial outcomes.

Nursing-related variable	Psychosocial outcome	Correlation coefficient (*r*/*β*)	*p*-value
Frequency of nursing consultations	HADS–anxiety	−0.34	< 0.001
Nursing psychosocial interventions	HADS–depression	−0.29	0.002
Patient–nurse communication quality	Global QoL (EORTC QLQ-C30)	+0.41	< 0.001
Nursing support score (composite)	HADS–anxiety	−0.32	< 0.001
Nursing support score (composite)	HADS–depression	−0.28	< 0.001
Nursing support score (composite)	Adaptive coping strategies	+0.26	< 0.001
Nursing support score (composite)	Global QoL (EORTC QLQ-C30)	+0.27	< 0.001

Correlation coefficients describing associations between nursing-related variables and psychosocial outcomes, including anxiety, depression, and quality of life. Negative coefficients indicate inverse associations, and positive coefficients indicate direct associations.

Depression scores were also evaluated as a psychosocial outcome. Patients receiving higher levels of nurse-led care demonstrated significantly lower depression scores compared with those receiving lower levels of nurse-led care (*p* < 0.05). Correlation analysis showed a significant negative association between nurse-led care and depression severity.

### 4.5 Disease stage and psychological distress

**Figure 4:**
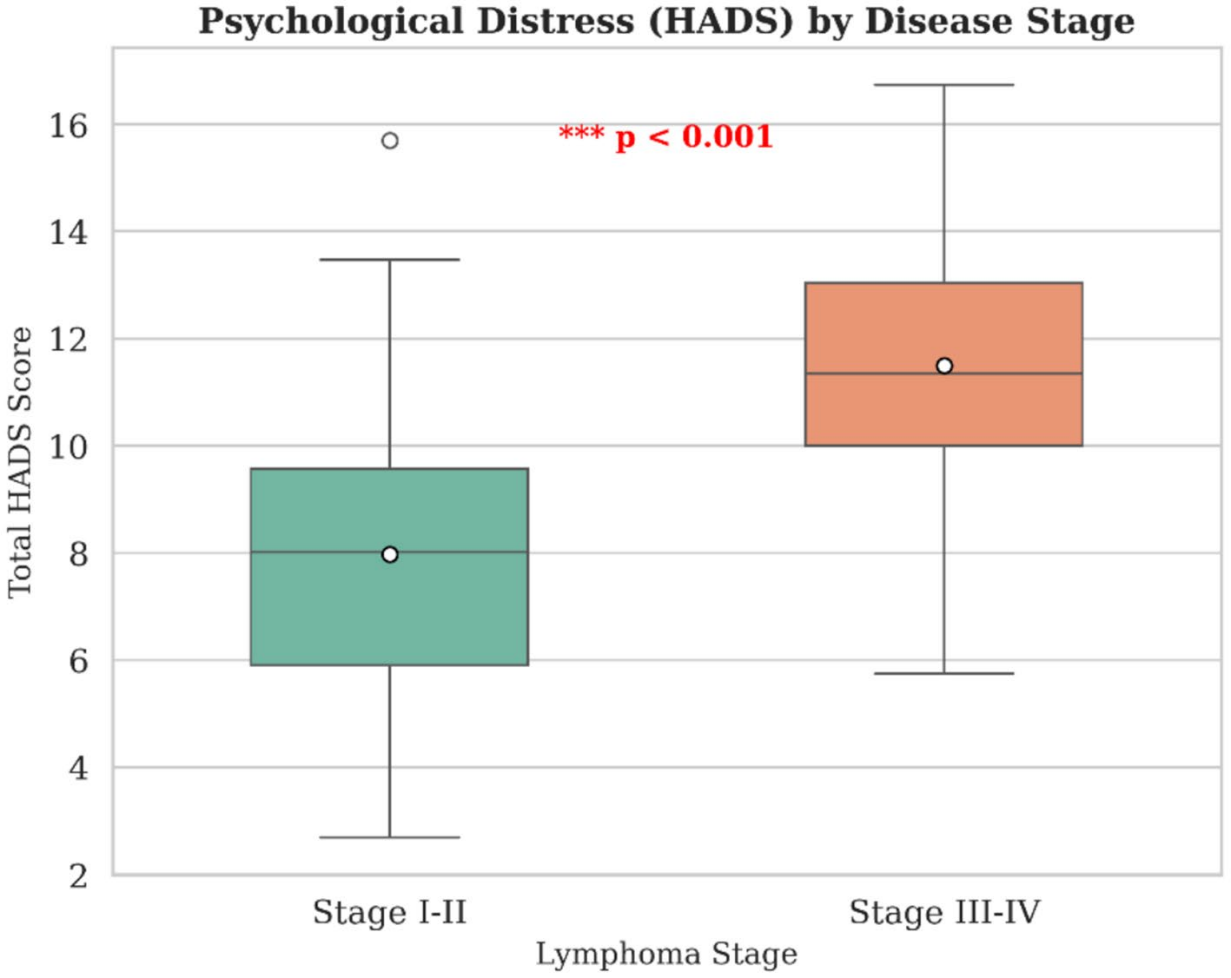
Comparison of psychological distress by disease stage. Box plots comparing total psychological distress (HADS score) between patients with early-stage (I–II) and advanced-stage (III–IV) lymphoma. The boxes represent interquartile ranges, the horizontal line indicates the median, and whiskers denote minimum and maximum values. ***p < 0.001.

Comparative analyses revealed marked differences in psychological distress by disease stage. Patients with advanced-stage lymphoma (stage III–IV) exhibited significantly higher total HADS scores compared to those with early-stage disease (stage I–II) (*p* < 0.001). This difference remained robust across anxiety and depression subscales and is visually illustrated in Figure 4. No significant sex-based differences in total HADS scores were observed.

### 4.6 Multivariable predictors of psychological distress

Multivariable regression analyses identified several independent predictors of psychological distress (Tables 6, 7**)**. After adjustment for sociodemographic and clinical variables, advanced disease stage (*β* = 0.38, *p* < 0.001) and greater reliance on avoidance-oriented coping strategies (*β* = 0.31, *p* = 0.001) were associated with increased distress. In contrast, higher levels of nursing psychosocial support were independently associated with lower overall psychological distress (*β* = −0.42, *p* < 0.001). Separate sensitivity analyses on the subscales confirmed that higher nursing support was independently associated with both lower anxiety and lower depression scores (anxiety: *β* = −0.39, *p* < 0.001; depression: *β* = −0.35, *p* < 0.001; data not shown in table).

**Table 6:** Multivariable regression analysis identifying independent predictors of psychological distress among patients with lymphoma.

Predictor variable	Model 1: unadjusted *β* (*p*-value)	Model 2: demographics-adjusted *β* (*p*-value)	Model 3: fully adjusted *β* (*p*-value)
Disease stage (III–IV)	0.41 (<0.001)	0.39 (<0.001)	0.38 (<0.001)
Avoidance-oriented coping	0.35 (<0.001)	0.33 (<0.001)	0.31 (0.001)
Nursing Support Score	−0.46 (<0.001)	−0.44 (<0.001)	−0.42 (<0.001)
Education level (higher)	−0.25 (0.006)	−0.23 (0.011)	−0.21 (0.022)
Age	—	0.07 (0.28)	0.06 (0.31)
Sex (male)	—	0.05 (0.34)	0.04 (0.41)
Lymphoma subtype (NHL)	—	—	0.09 (0.17)
Treatment modality	—	—	0.08 (0.19)

**Table 7:** Model fit statistics.

Statistic	Model 1	Model 2	Model 3
*R^2^*	0.42	0.49	0.56
Adjusted *R^2^*	0.41	0.47	0.54
Δ*R^2^*	—	0.07	0.07
F statistic	47.6	38.9	42.3
*p*-value (model)	<0.001	<0.001	<0.001

HADS, Hospital Anxiety and Depression Scale; *β*, standardized regression coefficient; NHL, non-Hodgkin lymphoma.

Model 1: Unadjusted model.

Model 2: Adjusted for age, sex, and education level.

Model 3: Fully adjusted for sociodemographic variables, clinical characteristics (lymphoma subtype, disease stage, and treatment modality), coping strategies, and nurse-led care.

All *β* values are standardized coefficients. Statistical significance set at *p* < 0.05.

Higher educational attainment also demonstrated a modest protective association (*β* = −0.21, *p* = 0.022). The fully adjusted model explained a substantial proportion of variance in psychological distress (adjusted *R^2^* = 0.54). Higher nursing support scores were moderately and negatively correlated with anxiety and depression, and positively correlated with global quality of life.

### 4.7 Mediation analysis

**Figure 5:**
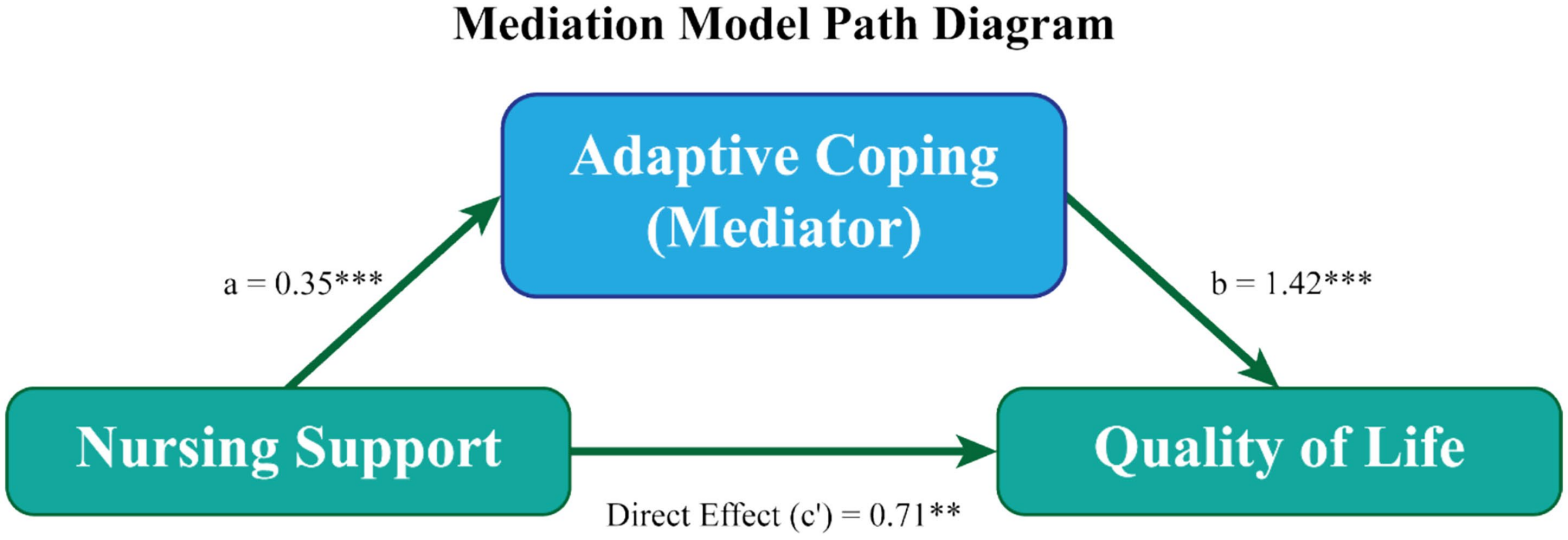
Mediation model of nurse-led care, adaptive coping, and quality of life. Path diagram illustrating the mediating role of adaptive coping strategies in the association between nursing support and quality of life. Standardized path coefficients are shown. The indirect effect was tested using bootstrapped confidence intervals. **p < 0.01, ***p < 0.001.

Mediation analysis was conducted to examine whether adaptive coping strategies mediated the relationship between nursing support and quality of life. Nurse-led care was positively associated with adaptive coping (path a = 0.35, *p* < 0.001), and adaptive coping was, in turn, positively associated with quality of life (path b = 1.42, *p* < 0.001). The direct effect of nursing support on quality of life remained significant after accounting for the mediator (c′ = 0.71, *p* < 0.01), indicating partial mediation. The indirect effect was statistically significant based on bootstrap confidence intervals, supporting the mediating role of adaptive coping (Figure 5).

### 4.8 Predictive performance of nursing support for anxiety

**Figure 6:**
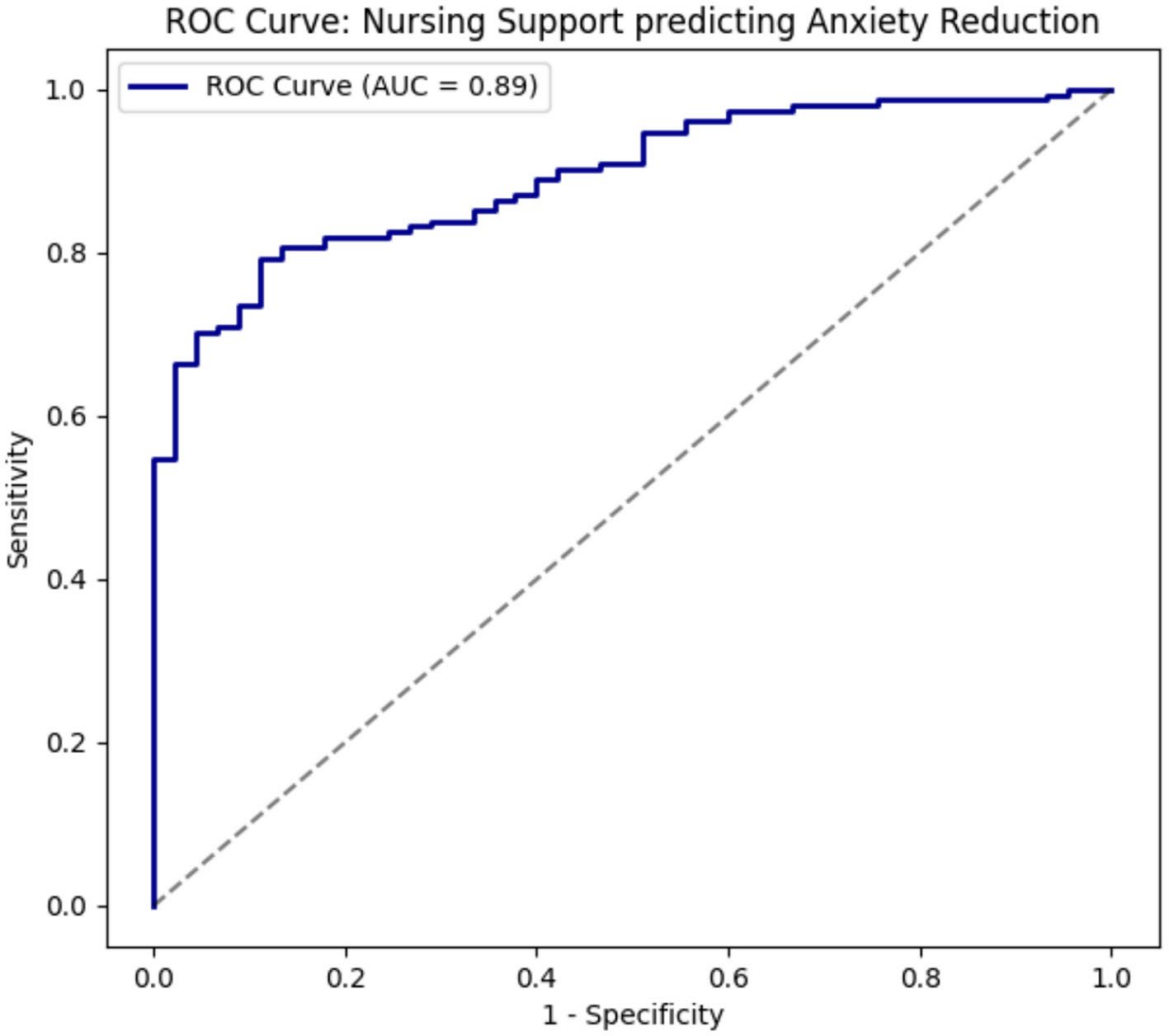
Receiver operating characteristic (ROC) curve for nursing support predicting clinically significant anxiety. ROC curve evaluating the discriminative ability of the nursing support score to identify patients with clinically significant anxiety (HADS-Anxiety ≥ 11). The area under the curve (AUC) indicates classification accuracy.

In exploratory ROC analysis, the Nursing Support Score showed good discriminative ability for identifying patients with clinically significant anxiety (HADS-Anxiety score ≥ 11), with an area under the curve (AUC) of 0.89 (95% CI: 0.84–0.94). The optimal cut-off value (determined by the Youden index) provided a favorable balance of sensitivity and specificity (Figure 6). These findings suggest that lower nursing support scores may help flag patients at higher risk of clinically relevant anxiety for further evaluation. However, the NSS is intended solely as a supplementary risk-stratification aid within routine nursing practice and should not be used as a substitute for validated psychological screening tools. Although the discriminative performance for clinically significant depression was also acceptable (AUC = 0.82), it was slightly lower than for anxiety; therefore, detailed ROC results are presented for anxiety only.

## 5 Discussion

This study provides a comprehensive evaluation of psychosocial burden, coping strategies, and quality of life among patients with lymphoma from a nurse-led care perspective. The findings demonstrate that psychological distress remains prevalent in this population and is strongly associated with disease stage, coping patterns, and the level of nursing support received. By integrating correlational, multivariable, mediation, and predictive analyses, the present study extends existing literature by elucidating the interconnected roles of nurse-led care and adaptive coping in shaping psychosocial outcomes.

The observed variability in nursing support should be interpreted in the context of adaptive clinical pathways, where care intensity is individualized. This variability represents a strength of patient-centered oncology nursing rather than a deviation from standardized care.

Previous studies support the beneficial role of nursing-focused interventions in reducing anxiety. A systematic review evaluating psychological nursing care in cancer patients reported that structured nursing interventions significantly reduced anxiety compared with routine care, highlighting the importance of targeted emotional support delivered by nurses (Zhu et al., 2022). Furthermore, nurse-led psychological counseling interventions have also been shown to significantly improve anxiety symptoms and patient quality of life, reinforcing the therapeutic value of nurse-led care approaches (Lyu et al., 2024).

The observed relationship may be explained by several mechanisms. Nurses spend more time with patients than other healthcare professionals, allowing them to identify emotional distress early and provide timely psychological support (Williams, 1997). Improved nurse–patient communication and empathetic interactions are central components of patient-centered nursing and have been associated with improved psychological outcomes (Chang et al., 2020). Additionally, individualized care planning and patient education may reduce uncertainty and improve coping ability, ultimately lowering anxiety levels (Pinho et al., 2021).

Our findings are also consistent with literature indicating that patient-centered care models improve psychological outcomes by addressing individual needs and promoting shared decision-making. Such approaches encourage active patient participation, improve satisfaction, and reduce emotional distress. Therefore, implementing structured nurse-led care protocols may represent an effective strategy to improve psychological outcomes in clinical settings.

A central contribution of this study lies in the robust association between nurse-led care and psychosocial outcomes. Higher levels of nurse-led supportive care were independently associated with lower anxiety and depression scores and with higher quality of life, even after adjustment for sociodemographic and clinical variables. These findings align with recent evidence from nurse-led models in lymphoma survivorship and hematological malignancies, where structured nursing coordination improves emotional functioning, adaptive coping, and overall well-being (Zhang et al., 2025; Choi et al., 2025).

Consistent with prior research in hematologic malignancies, patients in the present cohort exhibited moderate levels of anxiety and depression alongside impaired quality of life. These findings align with earlier reports indicating that lymphoma patients experience sustained psychological vulnerability due to diagnostic uncertainty, aggressive treatment regimens, and prolonged disease trajectories. Importantly, the observed distress levels were not uniform across the sample; patients with advanced-stage disease demonstrated significantly higher psychological burden compared with those in early stages. This stage-dependent gradient of distress likely reflects greater symptom burden, treatment complexity, and perceived threat to survival in advanced disease, reinforcing the need for stratified psychosocial screening in routine hematology care.

Coping strategies emerged as a critical psychosocial determinant. Adaptive coping mechanisms particularly acceptance and active coping were more frequently employed than maladaptive strategies, suggesting a degree of psychological adjustment within the cohort. Nevertheless, avoidance-oriented coping remained a significant independent predictor of heightened psychological distress in multivariable analyses. This finding is consistent with stress and coping theory, which posits that avoidance behaviors may provide short-term emotional relief but ultimately exacerbate distress by impeding effective problem-solving and emotional processing. The identification of avoidance coping as a risk factor highlights a potential target for nursing-led psychosocial interventions.

Coping strategies were aggregated into problem-focused, emotion-focused, and avoidance-oriented domains based on theoretical models rather than data-driven factor analysis in the present sample. This approach enhances comparability with prior oncology research. Although self-distraction and venting can serve adaptive functions in some contexts (e.g., short-term relief during acute treatment), they were grouped with other avoidance strategies in this study because they were positively associated with psychological distress in the current cohort and in previous lymphoma and cancer studies. Future research using person-centered or daily-diary designs may further elucidate the context-dependent utility of these strategies.

A central contribution of this study lies in the robust association between nurse-led care and psychosocial outcomes. Higher levels of nursing support were independently associated with lower anxiety and depression scores and with higher quality of life, even after adjustment for sociodemographic and clinical variables. These findings suggest that oncology nurses may contribute meaningfully to reducing psychosocial burden through sustained patient engagement, emotional support, and therapeutic communication. These findings underscore the pivotal role of oncology nurses in mitigating psychosocial burden through continuous patient engagement, emotional support, and therapeutic communication. Unlike physician encounters, nursing interactions are typically more frequent and sustained, positioning nurses as key agents in identifying distress and facilitating adaptive coping responses.

The observation that nearly half of the patients (45%) exceeded the clinical threshold for anxiety and more than one-third (37%) for depression aligns with previous reports in hematologic malignancies and reinforces the importance of systematic psychosocial evaluation in routine hematology nursing practice.

The mediation analysis suggests that adaptive coping strategies may partially account for the observed relationship between nursing support and quality of life. Nurse-led care was positively correlated with adaptive coping, which in turn was positively correlated with quality of life. The direct association between nursing support and quality of life remained significant after accounting for adaptive coping, consistent with a possible partial mediating role of adaptive coping. Adaptive coping partially mediated the relationship between nursing support and quality of life, indicating that nurse-led care may enhance well-being not only through direct supportive effects but also indirectly by fostering more effective coping strategies. This partial mediation suggests that while nursing support exerts an independent influence on quality of life, its impact is amplified when patients are supported in developing adaptive coping responses. These findings provide empirical support for conceptual models that position nursing care as a facilitator of psychological resilience rather than solely a provider of symptom management.

The ROC analysis adds a clinically relevant dimension to the findings by demonstrating that nursing support exhibits strong discriminative ability for identifying patients with clinically significant anxiety. Although nursing support should not be construed as a diagnostic tool, its high predictive accuracy suggests potential utility as a screening or risk stratification indicator within routine nursing assessments. This finding reinforces the value of systematically documenting nursing interactions and patient perceptions of care as part of comprehensive psychosocial evaluation.

The exploratory ROC analysis further demonstrated that the Nursing Support Score had strong discriminative capacity (AUC = 0.89) for identifying patients with clinically significant anxiety. This finding is clinically relevant because oncology nurses routinely interact with patients more frequently than other healthcare professionals and already document care encounters and interventions. A composite nursing support metric could therefore serve as a low-burden, practice-integrated signal to prompt more structured psychological assessment using validated instruments such as the HADS. We wish to emphasize that the NSS is not proposed as a formal screening tool and should not replace established psychometric instruments. Instead, it may function as an adjunctive risk-stratification indicator to help nursing teams prioritize patients who might benefit from deeper psychosocial evaluation or referral to specialized psycho-oncology services. Future prospective studies are needed to validate this application in diverse clinical settings.

From a clinical and nursing practice perspective, the results have several important implications. First, routine psychosocial screening using validated instruments such as the HADS should be integrated into standard nursing workflows, particularly for patients with advanced-stage disease. Second, nursing interventions could usefully incorporate strategies aimed at strengthening adaptive coping and reducing avoidance-oriented coping, with an emphasis on reducing avoidance behaviors and strengthening adaptive strategies such as acceptance and active problem-solving. Third, structured nurse-led care psychosocial programs may offer a feasible and cost-effective approach to improving quality of life in lymphoma patients without requiring extensive additional resources.

The present study was conducted in a single tertiary university hospital in Suzhou, China. Several cultural and systemic factors specific to the Chinese context should be considered when interpreting the findings. Chinese culture is predominantly collectivist, which may foster greater reliance on family support and emotional restraint in expressing psychological distress compared with more individualistic Western cultures (Lu et al., 2022; Kitayama and Park, 2007; Markus and Kitayama, 1991). This cultural orientation could influence both the reporting of anxiety and depression on self-report scales and the preferred coping strategies, particularly higher endorsement of acceptance and religious/spiritual coping. Additionally, the organization of oncology nursing in China characterized by relatively high nurse-to-patient ratios in tertiary centers and a strong emphasis on holistic supportive care and family involvement—may differ from nursing models in other countries. Consequently, the observed strong associations between nursing support and psychosocial outcomes may be partly amplified by these contextual features. While the core mechanisms linking nurse-led care, adaptive coping, and quality of life are likely universal, the magnitude of effects and optimal implementation strategies may vary across healthcare systems, cultural backgrounds, and resource settings. Future multi-center and cross-cultural studies are therefore needed to examine the transportability of these findings.

Several limitations should be acknowledged. The cross-sectional design precludes causal inference, and the observed associations should be interpreted as relational rather than directional. Data were collected from a single tertiary center, which may limit generalizability to other healthcare settings or cultural contexts. Psychosocial outcomes and coping strategies were assessed using self-report instruments, which are subject to reporting and social desirability biases. Additionally, although the nursing support score captured multiple dimensions of nursing care, it may not fully encompass the complexity and qualitative nuances of nurse–patient interactions.

Despite these limitations, the study possesses notable strengths, including a relatively large sample size, use of validated instruments, and a comprehensive analytical approach integrating mediation and predictive modeling. The explicit nurse-led framework enhances the translational relevance of the findings and supports their applicability to clinical practice.

Although the Nursing Support Score demonstrated good discriminative performance in ROC analysis, this was an exploratory finding. The score should be viewed as a potential risk-stratification support rather than a diagnostic or standalone screening instrument. Its clinical utility requires further validation in longitudinal and multi-center studies.

Additionally, the classification of certain Brief COPE subscales (particularly self-distraction and venting) as maladaptive remains subject to debate, as these strategies may be context or culture-dependent. Although sensitivity analyses supported the robustness of our findings, alternative classification approaches could yield slightly different results.

The application of upper limits and bounded ranges in the Nursing Support Score, while methodologically intended to reduce the influence of extreme values and improve comparability, may attenuate variability among patients receiving very high levels of nursing care. Although sensitivity analyses supported the robustness of findings, future studies using longitudinal designs may further refine these measurements.

In conclusion, this study demonstrates that psychosocial distress remains a significant concern among patients with lymphoma and is influenced by disease stage, coping strategies, and nurse-led care. Higher nursing support was associated with lower psychological distress and better quality of life and enhancing quality of life, in part through its association with adaptive coping. These findings underscore the importance of integrating structured psychosocial assessment and coping-focused interventions into routine oncology nursing practice and provide a foundation for future longitudinal and interventional studies aimed at optimizing supportive care for lymphoma patients.

## 6 Conclusion

This study demonstrates that patients with lymphoma experience a substantial psychosocial burden, with 45% and 37% of participants reporting clinically significant anxiety and depression, respectively, alongside impaired quality of life. Psychological distress was not uniformly distributed across the cohort and was significantly influenced by disease stage, coping patterns, and the level of nurse-led care received. Patients with advanced-stage disease and those who relied more heavily on avoidance-oriented coping strategies were particularly vulnerable to heightened psychological distress.

A key finding of this study is the central role of nurse-led care in shaping psychosocial outcomes. Higher levels of nursing support were independently associated with lower psychological distress and improved quality of life, even after adjustment for relevant sociodemographic and clinical factors. Moreover, adaptive coping strategies partially mediated the relationship between nursing support and quality of life, suggesting that findings are consistent with the possibility that nursing support is linked to better well-being both directly and indirectly through its association with more adaptive coping responses by facilitating more effective coping responses.

From a clinical perspective, these findings highlight the importance of integrating routine psychosocial screening and coping-focused assessments into standard hematology nursing practice. Structured nursing interventions that emphasize therapeutic communication, emotional support, and the promotion of adaptive coping strategies may represent an effective approach to mitigating psychosocial distress among lymphoma patients, particularly those with advanced disease.

In summary, the present study provides evidence that nurse-led care constitutes a critical component of comprehensive supportive oncology care. By addressing both psychological distress and coping processes, oncology nurses can play a pivotal role in enhancing the overall well-being and quality of life of patients with lymphoma. Future longitudinal and interventional studies are warranted to further elucidate causal pathways and to evaluate the effectiveness of targeted nursing-led psychosocial interventions.

## Data Availability

The original contributions presented in the study are included in the article/supplementary material, further inquiries can be directed to the corresponding author.
